# Biological Treatment for Uncontrolled Chronic Rhinosinusitis with Nasal Polyps: Preliminary Real-World Results from a Tertiary Medical Center

**DOI:** 10.3390/jcm12113671

**Published:** 2023-05-25

**Authors:** Reut Book, Shalom Eligal, Yuval Tal, Ron Eliashar

**Affiliations:** 1 Department of Otolaryngology/Head and Neck Surgery, Hadassah Medical Center, Faculty of Medicine, Hebrew University, Jerusalem 91120, Israel; reutbook@gmail.com; 2 Hadassah Medical Center, Faculty of Medicine, Hebrew University, Jerusalem 91120, Israel; shlomielig@gmail.com; 3 Allergy and Clinical Immunology Unit, Department of Medicine, Hadassah Medical Center, Faculty of Medicine, Hebrew University, Jerusalem 91120, Israel; talyuv@gmail.com

**Keywords:** chronic rhinosinusitis, nasal polyposis, biological treatment, monoclonal antibodies, dupilumab, benralizumab, omalizumab, SNOT-22, endoscopic sinus surgery, Type-2 inflammation

## Abstract

The efficacy of biological treatment for severe uncontrolled chronic rhinosinusitis with nasal polyps (CRSwNP) has recently been demonstrated through double-blinded clinical trials. The aim of this study was to provide preliminary real-world experience regarding biological therapy for uncontrolled CRSwNP. The records of patients who received biological treatment in a tertiary medical center between the years 2019 to 2022 were retrospectively reviewed. Patients included in this study were eligible for biological treatment according to the EPOS 2020 criteria. Among patients who had their first follow-up visit <6 months from the treatment initiation, the Sino-Nasal Outcome Test 22 Questionnaire (SNOT-22) score had decreased by 22% (*p* = 0.01) and the nasal polyp score (NPS) had decreased by 48% (*p* = 0.05). Among patients who had their first follow-up visit ≥6 months from treatment initiation, the SNOT-22 score had decreased by 40% (*p* = 0.03) and the NPS had decreased by 39% (*p* = 0.1). The number of patients who needed systemic steroid treatment had decreased by 68% (*p* < 0.0001), and the number of patients who needed endoscopic sinus surgery had decreased by 74% (*p* < 0.0001). These findings correspond with the improvement of clinical symptoms observed in prior randomized clinical trials, thus showing the effectiveness of biologic medications in the treatment of severe CRSwNP in a real-life setting. Although further cohort studies are warranted, our study also suggests evaluating patients at follow-up visits mainly by quality-of-life aspects and investigating longer dosing intervals of dupilumab.

## 1. Introduction

Chronic rhinosinusitis (CRS) in adults is defined as the presence of ≥2 symptoms, 1 of which should be either nasal obstruction or nasal discharge (anterior/posterior nasal drip) ± facial pain/pressure ± reduction or loss of smell, for ≥12 weeks. Chronic rhinosinusitis with nasal polyps (CRSwNP) is characterized by bilateral polyps, endoscopically visualized in the middle and/or superior meatus [1].

The prevalence of CRSwNP is estimated at around 2.5% [2]. CRSwNP has a significant negative impact on the patients’ quality of life. Affected patients often present with other comorbidities, such as asthma, allergic rhinitis, and intolerance to non-steroidal anti-inflammatory drugs (NSAIDs), contributing to the severity of the disease [1,2,3]. Despite surgery [Endoscopic Sinus Surgery (ESS)] and ongoing intranasal corticosteroid (INCS) and/or systemic corticosteroid therapy, the estimated number of patients remaining symptomatic with incomplete disease control is high, verging around 40–50% of cases [4,5].

CRSwNP has been classified as a Type-2 inflammatory disease [1]. As such, it is driven by the key cytokines Interleukin (IL)-4, IL-5, and IL-13 [5]. In addition, IgE is also thought to perform a central role in CRSwNP pathogenesis by activating and generating degranulation of mast cells and basophils, thus causing activation of other immune cells, including Th2 cells and eosinophils [6]. Due to the main characteristics of CRSwNP, biological treatments with monoclonal antibodies have been recently introduced as an optional treatment for patients with poor disease control.

Dupilumab, an anti-IL-4Rα monoclonal antibody inhibiting IL-4 and IL-13 signaling, was the first biological drug approved in 2019 by the FDA as an adjunct therapy to intra-nasal corticosteroids in adult patients suffering from CRSwNP [7,8]. Dupilumab demonstrates substantial disease modifying properties leading to considerable symptom improvement in patients with difficult-to-control CRSwNP [8,9,10].

IL-5 performs an important role in promoting eosinophil proliferation, activation, and survival. Different anti-IL-5 receptor monoclonal antibodies showed effectiveness in CRSwNP [11]. Benralizumab, an anti-IL5Rα antibody, was shown to reduce nasal polyp score (NPS), decrease nasal blockage, and reduce difficulty with sense of smell when added to mainstay treatment in patients with CRSwNP [12]. Mepolizumab, a humanized anti-IL-5 monoclonal antibody, presented an effective add-on treatment option to the standard of care in patients with recurrent, refractory severe CRSwNP and was approved in 2021 by the FDA for this label [13].

Omalizumab, a humanized anti-IgE monoclonal antibody, binds free IgE, thus blocking the interaction of IgE with its high-affinity receptor on mast cells and basophils. Omalizumab received FDA approval for severe allergic asthma in 2003 [14]. In 2020, omalizumab was approved for the treatment of CRSwNP patients after demonstrating beneficial effects [6,11].

Despite this data, there are very limited reports regarding the real-world efficacy of currently available biologic treatments and treatment-related outcomes in comparison to clinical trial results. The aim of this study is to present preliminary results of real-world data from a rhinology clinic in a tertiary medical center treating CRSwNP patients. Since the main goal of biological therapy for CRSwNP is the improvement in quality of life (QoL), which is often assessed through patients’ subjective statements and by validated QoL questionnaires, such as the Sino-Nasal Outcome Test 22 (SNOT-22) [15]. We chose to focus mainly on the SNOT-22 and on subjective statements rather than on radiologic or endoscopic findings based on evidence regarding no correlation between symptoms’ relief and radiologic or endoscopic findings [16,17,18,19,20]. In addition to our preliminary study, a national multicenter study is expected to commence soon.

## 2. Materials and Methods

The study is a retrospective observational study involving patients who received biological treatment for CRSwNP from 2019 to 2022 at a tertiary medical center. The study was approved by the local Helsinki Committee [0361-22-HMO].

All patients suffered from CRSwNP and met the EPOS 2020 criteria for being eligible for biological treatment [1]. As such, they all received the mainstay treatment for CRSwNP before biological treatment initiation, including INCS, systemic corticosteroids, and ESS (unless there were contraindications to systemic corticosteroids and/or to ESS). All patients had evidence of Type 2 inflammation based on either laboratory results (high levels of blood or tissue eosinophils; total IgE > 100) or clinical findings (allergy with a positive skin prick test; presence of late-onset asthma; NSAIDs intolerance). All were treated with one of the following biologic medications: dupilumab, omalizumab, benralizumab, or mepolizumab. Benralizumab was given by Pulmonologists for its approved indication of severe eosinophilic asthma. Treatment was given either at a clinic or at home.

Data on the patient status at presentation and in every follow-up visit was retrieved from the patients’ electronic medical records. It included demographics, clinical and background features, NPS, SNOT-22 questionnaire, and laboratory data when available. SNOT-22 is a validated questionnaire used for measuring the impact of CRS on the patient’s QoL. It may also be used for measuring the outcome of surgical interventions [21,22]. Nasal polyp burden was endoscopically evaluated using the nasal polyp scoring system: the nasal cavity was bilaterally evaluated, and each side was scored in a range from 0 to 4 (0  =  no polyps, 1  =  polyps limited to the middle meatus, 2  =  presence of multiple polyps in the middle meatus, 3  =  extension of polyps beyond the middle meatus, 4  =  complete nasal cavity obstruction by nasal polyps) [23]. NPS was recorded as the sum of scores of both nasal cavity sides, ranging from 0 to 8.

Patients who did not come to their follow-up visit were contacted by phone. They were asked open-ended questions about their sino-nasal-related QoL and were investigated about the side effects of the biological treatment.

To test the association between two categorical variables, the chi-square test was applied. The comparison of a quantitative variable was carried out using the paired *t*-test. To simultaneously assess the effect of several variables on the dependent variable, the multivariate logistic regression model was used.

## 3. Results

Forty-three patients were considered for biological treatment during the study period. Out of those, 38 patients were able to attain insurance coverage for the recommended therapy. The patients’ baseline characteristics are presented in Table 1. The mean age was 51.55 (range 22–81), and 18 (47%) of them were females. 36 (95%) patients had at least one ESS prior to the biological treatment initiation (mean: 2.13 ± 1.33), with an average of 29.6 ± 29.7 months since the latest surgery preceding treatment induction. The baseline SNOT-22 score and NPS were 62.96 ± 25.42 and 2.1 ± 1.3, respectively.

Twenty-five (65%) patients attended the Rhinology Clinic for follow-up visits. Table 1 provides a comparison of baseline data between patients who attended the follow-up visits and those who did not. No statistically significant differences were observed between the two groups, including in relation to SNOT-22 score (56.21 ± 28.33 vs. 71.54 ± 19.04, *p* = 0.13) and NPS (1.87 ± 1.42 vs. 2.5 ± 0.9, *p* = 0.14). Given that the time from treatment initiation to the first follow-up visit varied between patients, we divided these 25 patients into two groups: Group 1 consisted of patients who had their first follow-up visit <6 months from treatment initiation; Group 2 was composed of patients who had their first follow-up visit ≥6 months from treatment initiation. First follow-up visits of patients in Group 2 did not exceed 12 months. Of these 25 patients, 16 (64%) were treated with dupilumab, 8 patients (32%) were treated with an anti-IL-5 agent (benralizumab/mepolizumab), and 1 patient (4%) was treated with omalizumab. Dupilumab was taken in 2 weeks intervals, with the exception of 2 patients who received every 3 weeks and 2 patients who received it every 4 weeks. Mepolizumab and Omalizumab were taken every 4 weeks. Benralizumab was initially taken every 4 weeks and then every 8 weeks.

**Figure 1:**
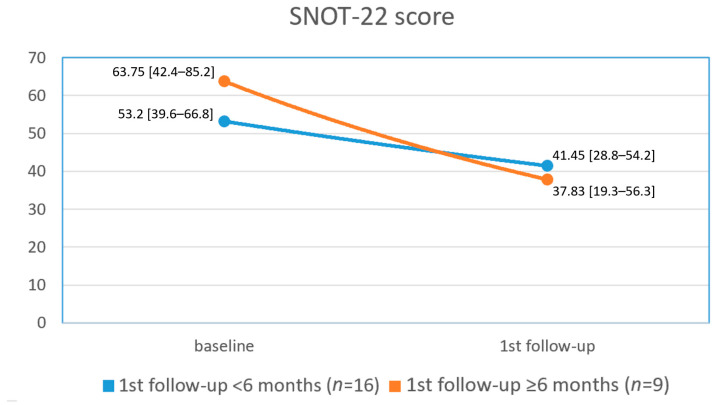
Mean [95% confidence interval] SNOT-22 scores at baseline and at the first follow-up visit in both groups.

**Figure 2:**
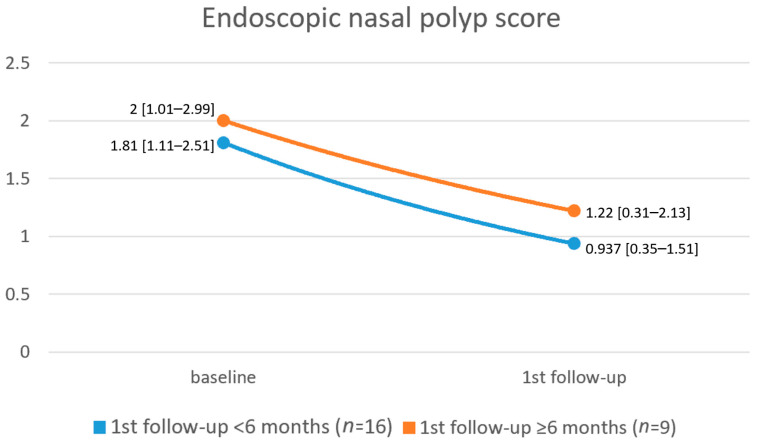
Mean [95% confidence interval] endoscopic nasal polyp score at baseline and at the first follow-up visit in both groups.

Patients in Group 1 had a mean baseline SNOT-22 score and NPS of 53.2 ± 27.71 and 1.81 ± 1.42, respectively. At the first follow-up visit (<6 months from treatment initiation), the mean SNOT-22 score was decreased by 22% to 41.45 ± 25.87 (*p* = 0.01), and the NPS was decreased by 48% to 0.937 ± 1.18 (*p* = 0.05). Results of SNOT-22 scores among patients in Group 2 (first follow-up visit ≥ 6 months from treatment initiation) demonstrated an even more significant decline in SNOT-22 scores at the first follow-up visit, with a diminution by 40% from 63.75 ± 32.7 at baseline to 37.83 ± 28.38 (*p* = 0.03). In this group, the NPS decreased by 39% from 2 ± 1.51 to 1.22 ± 1.39 (*p* = 0.1) [Figure 1 and Figure 2].

Patients who received dupilumab with extended intervals between doses (every 3 or 4 weeks) showed comparable results, with a 43% decrease in SNOT-22 score (from 55.66 ± 7.76 to 31.75 ± 9.81) and an 87% decrease in NPS (from 2 ± 1.15 to 0.25 ± 0.5). Over a twelve-month follow-up period, the number of patients who needed systemic (IM/PO) steroid treatment was decreased by 68% (from 25 to 8, *p* < 0.0001). Similarly, the number of patients who needed revision ESS during the follow-up period declined by 74% (from 23 to 6, *p* < 0.0001). We considered an improvement of greater than nine points in the SNOT-22 score as the minimum clinically significant improvement [24]. We utilized two multivariate analysis models to examine variables associated with clinically significant improvement in SNOT-22 score. Model #1 integrated the absolute count of blood eosinophils, while Model #2 incorporated the eosinophil percentage. The corresponding results are presented in Table 2 and Table 3.

**Figure 3:**
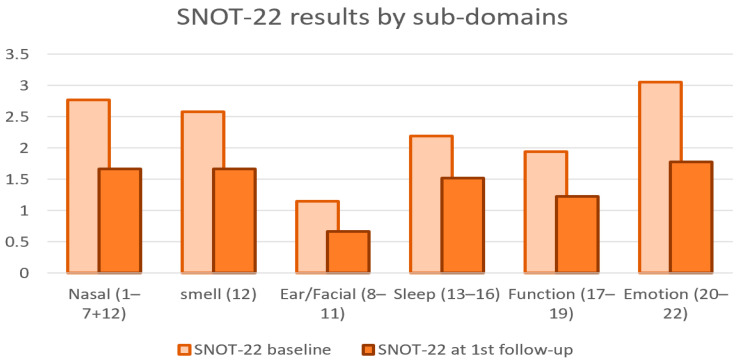
SNOT-22 sub-domains scores before and after treatment.

Focusing on relevant sub-domains of SNOT-22 scores [Figure 3], the ear/facial, nasal, and emotion domains demonstrated the most significant improvement, with 42% (*p* = 0.02), 40% (*p* = 0.01), and 41% (*p* = 0.002) decline, respectively. The sleep domain showed the lowest improvement rate, with a 30% (*p* = 0.05) decline. The smell and function domains had a 35% (*p* = 0.07) and 37% (*p* = 0.04) decline, respectively.

During each follow-up visit, the patients were screened for potential side effects related to the biological treatment. Of the 25 patients who attended the follow-up visits, 21 patients (84%) had no side effects. One patient reported a foul urine smell and on joint pain from dupilumab at the first follow-up visit 6 months after treatment initiation. Another patient reported slight swelling in the injection site at a 12 month visit and another skin erythema at a 3 month follow-up visit. Lastly, one patient reported an eye itch 6 months after treatment initiation. All were mild and temporary and did not prevent the patients from continuation of treatment. There were no reported side effects from the other biological agents at the time of follow-up at the rhinology clinic; however, one patient had reported side effects from omalizumab and from mepolizumab received before presenting to our clinic, such as salivary glands swelling, skin itch, and a metal taste.

Patients who did not arrive for their follow-up visit were contacted by phone. Additionally, one was very satisfied with the treatment and expressions, such as “the drug has saved my life”, were offered repeatedly by the patients. One patient did not benefit from treatment with Dupilumab and therefore underwent another ESS two years ago, after which she did not continue treatment with Dupilumab since she felt well and was bothered by the need to take it every other week, and since it was too expensive. However, she still feels very well even without Dupilumab. None had any side effects. When asked why they missed follow-up, most patients mentioned that they felt so good that they did not bother traveling to the hospital. Some mentioned that they did not receive approval from their Health Maintenance Organization (HMO).

## 4. Discussion

CRSwNP is a relatively common medical disorder characterized by both obstructive nasal symptoms and endoscopically visualized bilateral nasal polyps [1]. The mainstay of treatment for CRSwNP is based on either standard local therapeutic treatment, such as INCS, or, in more severe and non-responsive cases, advanced treatment with systemic corticosteroids and ESS [25]. However, there are patients with severe uncontrolled CRSwNP who do not respond to medical and/or surgical treatment and still experience symptoms with disease persistence [26]. Given that the basic inflammatory pathogenesis of CRSwNP has been classified as a Type-2 inflammatory disease, driven by the key cytokines IL-4, IL-5, and IL-13, and IgE antibody, biological treatments with monoclonal antibodies have been introduced as an optional treatment for patients with poor disease control [5]. Two double-blind, placebo-controlled clinical trials demonstrated the efficacy and safety of dupilumab in severe uncontrolled CRSwNP. Patients obtained significant improvements in nasal obstruction symptoms and in NPS [8].

In this study, we presented our preliminary experience regarding the effectiveness of biological treatment in patients with severe uncontrolled CRSwNP in a real-life setting. Our findings demonstrate a 22% decline in SNOT-22 score (from 53.2 ± 27.71 to 41.45 ± 25.87, *p* = 0.01) and a 48% decline in NPS (from 1.81 ± 1.42 to 0.937 ± 1.18, *p* = 0.05) among patients who had their first follow-up visit <6 months from treatment initiation. Furthermore, we demonstrated a 40% decline in SNOT-22 score (from 63.75 ± 32.7 to 37.83 ± 28.38, *p* = 0.03) and a 39% decline in NPS score (from 2 ± 1.51 to 1.22 ± 1.39, *p* = 0.1) among patients who had their first follow-up visit ≥6 months from treatment initiation. The disparity in baseline levels of SNOT-22 and NPS between the two groups is not readily discernible since the grouping was based on follow-up time rather than disease severity. However, we propose a hypothesis that patients with more severe initial disease conditions may have experienced a more pronounced improvement and benefit from the biological treatment. Consequently, these patients may have been less inclined to promptly return for follow-up visits as they perceived significant improvements in their well-being. It is crucial to consider this aspect when interpreting the findings and recognizing the potential influence of disease severity on patients’ motivation to seek continued care.

Interestingly, regarding the SNOT-22 sub-domains, we found that the sleep domain had the lowest improvement rate, with a 30% (*p* = 0.05) decline. This finding might be attributed to the fact that most of the follow-up meetings took place during the COVID-19 era, which had a domestic effect of poor sleep and increased stress in the general population. The ear/facial, nasal, and emotion domains showed the most significant improvement, with 42% (*p* = 0.02), 40% (*p* = 0.01), and 41% (*p* = 0.002)decline, respectively. The smell and function domains had a 35% (*p* = 0.07) and 37% (*p* = 0.04) decline, respectively.

Additionally, we found that during follow-up visits, the number of patients who needed systemic steroid treatment and/or ESS had significantly declined, with a decrease of 68% and 74% compared to baseline, respectively (*p* < 0.0001). These findings provide additional reinforcement to the seemingly positive effect following biological treatment, including well-controlled symptoms and disease severity in these severe uncontrolled CRSwNP patients.

Our study design primarily focused on measuring improvements in QoL of patients suffering from CRSwNP, as reflected in both SNOT-22 scores and subjective expressions by our patients. This is due to evidence that improvement in disease-specific health-related QoL outcomes does not necessarily correlate with endoscopic examination [19], eosinophilic status [16], nasal polyp status [18], histopathology [17], or radiologic score [20]. Therefore, we included patients who missed their follow-up visits as well, all of whom expressed significant satisfaction from their symptoms’ relief and improvement in QoL. They also reported no side effects and, furthermore, felt so well that they did not bother to arrive at their follow-up visits. Our patient-centered approach, prioritizing improvement in QoL and symptom relief during follow-up visits, has the potential to yield economic and safety benefits. This is in contrast to a paradigm relying heavily on radiologic scores, which can involve multiple CT scans and may increase both radiation exposure and costs.

Our study also addresses the issue regarding the interval between the doses of dupilumab. The standard protocol suggests a dose every two weeks [15]. During our study, four patients were administrated dupilumab with dose intervals every three or even every four weeks, with comparable outcomes in terms of SNOT-22 scores and NPS. This might suggest a potential for treatment with longer intervals between doses, thus reducing discomfort and costs and increasing the chances of receiving insurance coverage. Our experience, along with others, shows that insurance companies do not provide coverage for dupilumab in more than 10% of patients [9]. Further studies are warranted in order to investigate longer dosing intervals.

Our findings suggest that all biological medications (dupilumab, omalizumab, benralizumab, and mepolizumab) seem to have good outcomes in disease control and symptom improvement. A meta-analysis by Shiru Cai et al. suggested that dupilumab exhibits the best efficacy for the treatment of CRSwNP [27].

Recently, initial real-world evidence of biological treatment efficacy for severe uncontrolled CRSwNP has been presented. Kilty et al., in their series of 43 patients who started dupilumab therapy, demonstrated a decrease of more than 50% in SNOT-22 score at 16 weeks from treatment induction [9]. This improvement was maintained and demonstrated during the 28 weeks and 1-year follow-ups. These results correspond with our data, demonstrating a consistent decline in SNOT-22 score over time. Our data, in addition, demonstrate a similar substantial decline in NPS and compare findings with other biological medications as well (e.g., omalizumab and benralizumab). Similar findings were demonstrated by De Corso et al., who showed a decline in SNOT-22 score and a reduction in nasal eosinophil infiltrate, a decrease in the need for ESS and oral corticosteroids, and improved control of associated comorbidities, such as chronic eosinophilic otitis media and bronchial asthma [10]. These findings resemble our results, showing improvement in QoL and a substantial decrease in the need for both systemic steroid treatment and ESS. Haxel et al., in their series of 70 patients, demonstrated clinical improvement in NPS, QoL, and olfactory function, following treatment with either dupilumab or omalizumab [28]. Our findings correspond to these results and, furthermore, demonstrate the effectiveness of additional biological medications as well (benralizumab and mepolizumab).

A comparison between ESS and dupilumab treatment was presented by Dharmarajan et al., who concluded that patients treated with ESS had a greater reduction in polyp burden compared to patients treated with dupilumab [29]. Nevertheless, patients treated with dupilumab reported greater improvement in olfaction and a more substantial decrease in cough, postnasal drainage, and thick nasal drainage. These findings suggest that dupilumab has greater effectiveness in terms of nasal symptom relief compared to ESS. In our institution, a comparison between ESS and biological treatment would not be applicable since, according to the EPOS 2020 criteria, indications for biological treatment include prior ESS as a mandatory criterion [1]. All patients in our study, unless contra-indicated, had, therefore at least one ESS prior to treatment initiation. Nevertheless, the substantial reduction in the severity of nasal symptoms demonstrated in Dharmarajan et al. series following dupilumab treatment correlates with our findings, showing a reduction of 40% in the SNOT-22 nasal subdomain score. Interestingly, Dharmarajan et al. findings could potentially explain our findings regarding the less continuous improvement in NPS over time (48% decrease rate in Group 1 vs. 39% decrease rate in Group 2) in contrast to the SNOT-22 score improvement rate (22% in Group 1 vs. 40% in Group 2). Moreover, these findings reinforce the paradigm discussed above that endoscopic or radiologic status does not necessarily correlate with symptom relief and improvement in QoL.

There are limitations to our study. First, the retrospective nature of the study may introduce inherent biases. Second, the relatively small sample size limited our ability to establish statistical significance for all the outcomes examined, resulting in significant findings only within a subset of our results. However, we aimed to provide preliminary real-world results regarding biological treatment. Presenting the clinical improvement rate following biological treatment should be suitable for this goal. Third, the time from treatment initiation to the first follow-up visit varied between patients. To overcome this issue, we divided the patients into two distinct groups based on the time of the first follow-up and demonstrated the improvement rates of each group separately. We aim to continue with a multicenter national study that will provide data on a much bigger set of patients with longer follow-up times.

## 5. Conclusions

Our real-world findings demonstrate significant improvement in both QoL parameters and in nasal polyp burden after biological treatment initiation. Furthermore, our study suggests that follow-up visits may focus on QoL parameters. Additionally, our preliminary results suggest that longer intervals between doses of dupilumab could possibly be considered for reducing discomfort and costs and increasing the chances of receiving insurance coverage. Further cohort studies are warranted to provide additional data and evidence regarding real-world effectiveness of biological treatment for severe uncontrolled CRSwNP.

## Figures and Tables

**Table 1:** Patients’ baseline characteristics.

Demographics, Laboratory Findings, Clinical Features and Background	Whole Sample (*n* = 38)	Attended the Follow-Up Visits (*n* = 25)	Did Not Attend Follow-Up Visits (*n* = 13)
Age, mean (range)	51.55 (22–81)	55.28 (22–81)	44.38 (30–73)
Gender (male:female)	20:18	13:12	7:6
Samter’s triad	9 (23%)	8 (32%)	1 (7%)
Asthma	34 (89%)	24 (96%)	10 (76%)
Allergic status	25 (65%)	18 (72%)	7 (53%)
Antihistamine/Montelukast usage	18 (47%)	13 (52%)	5 (38%)
Steroid nasal spray usage	27 (71%)	20 (80%)	7 (53%)
Steroid inhaler usage	31 (82%)	23 (92%)	8 (61%)
Blood Eosinophils%, mean ± SD	8.26 ± 4.53	8.33 ± 5.26	8.12 ± 2.92
Blood Eosinophils#, mean ± SD	0.76 ± 0.92	0.57 ± 0.45	1.14 ± 1.46
Total IgE, mean ± SD	310 ± 426	400 ± 545	158 ± 214
Systemic steroid treatment	38 (100%)	25 (100%)	13 (100%)
Number of prior Endoscopic Sinus Surgeries, Mean ± SD	2.13 ± 1.33	2.12 ± 1.30	2.15 ± 1.46
Months since last surgery, Mean ± SD	29.6 ± 29.7	25.45 ± 20.50	37.9 ± 42.6
Endoscopic nasal polyp score, Mean ± SD	2.1 ± 1.3	1.87 ± 1.42	2.5 ± 0.9
SNOT-22 score, Mean ± SD	62.96 ± 25.42	56.21 ± 28.33	71.54 ± 19.04

**Table 2:** Model #1 (absolute count of blood eosinophils) of multivariate logistic regression of variables associated with clinically significant improvement (>9) in SNOT-22 score.

Variable	95% Confidence Interval	Odds Ratio	*p* Value
Age	−0.3–0.16	0.93	0.32
Gender	−2.26–4.4	2.9	0.3
Samter’s triad	−2.71–2.69	0.98	0.98
Asthma	−9.05–12.0	4.38	0.6
Antihistamine/Montelukast usage	−3.3–2.89	0.81	0.8
Steroid nasal spray usage	−2.24–3.84	2.21	0.37
Steroid inhaler usage	−4.53–4.75	1.12	0.92
Blood Eosinophils#	−1.39–2.27	1.55	0.4
Number of prior endoscopic sinus surgeries	−1.54–1.86	1.17	0.72

**Table 3:** Model #2 (eosinophil percentage) of multivariate logistic regression of variables associated with clinically significant improvement (>9) in SNOT-22 score.

Variable	95% Confidence Interval	Odds Ratio	*p* Value
Age	−0.13–0.02	0.94	0.09
Gender	−0.42–1.97	2.16	0.1
Samter’s triad	−1.07–0.98	0.95	0.87
Asthma	−2.68–5.42	3.93	0.28
Antihistamine/Montelukast usage	−1.28–0.99	0.86	0.64
Steroid nasal spray usage	−0.46–1.71	1.86	0.13
Steroid inhaler usage	−1.75–1.72	0.98	0.97
Blood Eosinophils%	−0.01–0.09	1.04	0.09
Number of prior endoscopic sinus surgeries	−0.55–0.77	1.11	0.55

## Data Availability

Not applicable.
